# Intracellular biliverdin dynamics during ferroptosis

**DOI:** 10.1093/jb/mvae067

**Published:** 2024-09-28

**Authors:** Kazuma Nakajima, Hironari Nishizawa, Guan Chen, Shunichi Tsuge, Mie Yamanaka, Machi Kiyohara, Riko Irikura, Mitsuyo Matsumoto, Kozo Tanaka, Rei Narikawa, Kazuhiko Igarashi

**Affiliations:** Department of Biochemistry, Tohoku University Graduate School of Medicine, 2-1 Seiryo-machi, Aoba-ku, Sendai, Miyagi 980-8575, Japan; Department of Biochemistry, Tohoku University Graduate School of Medicine, 2-1 Seiryo-machi, Aoba-ku, Sendai, Miyagi 980-8575, Japan; Department of Molecular Oncology, Institute of Development, Aging and Cancer, Tohoku University, 4-1 Seiryo-machi, Aoba-ku, Sendai, Miyagi 980-8575, Japan; Department of Biochemistry, Tohoku University Graduate School of Medicine, 2-1 Seiryo-machi, Aoba-ku, Sendai, Miyagi 980-8575, Japan; Department of Biochemistry, Tohoku University Graduate School of Medicine, 2-1 Seiryo-machi, Aoba-ku, Sendai, Miyagi 980-8575, Japan; Gladstone Institute of Neurological Disease, Gladstone Institutes, 1650 Owens street, San Francisco, CA 94158, USA; Department of Biochemistry, Tohoku University Graduate School of Medicine, 2-1 Seiryo-machi, Aoba-ku, Sendai, Miyagi 980-8575, Japan; Department of Biochemistry, Tohoku University Graduate School of Medicine, 2-1 Seiryo-machi, Aoba-ku, Sendai, Miyagi 980-8575, Japan; Department of Biochemistry, Tohoku University Graduate School of Medicine, 2-1 Seiryo-machi, Aoba-ku, Sendai, Miyagi 980-8575, Japan; Center for Regulatory Epigenome and Diseases, Tohoku University Graduate School of Medicine, 2-1 Seiryo-machi, Aoba-ku, Sendai, Miyagi 980-8575, Japan; Department of Molecular Oncology, Institute of Development, Aging and Cancer, Tohoku University, 4-1 Seiryo-machi, Aoba-ku, Sendai, Miyagi 980-8575, Japan; Department of Biological Sciences, Graduate School of Science, Tokyo Metropolitan University, 1-1 Minami-Ohsawa, Hachioji, Tokyo 192-0397, Japan; Department of Biochemistry, Tohoku University Graduate School of Medicine, 2-1 Seiryo-machi, Aoba-ku, Sendai, Miyagi 980-8575, Japan; Center for Regulatory Epigenome and Diseases, Tohoku University Graduate School of Medicine, 2-1 Seiryo-machi, Aoba-ku, Sendai, Miyagi 980-8575, Japan

**Keywords:** BACH1, biliverdin, cyanobacteriochrome, ferroptosis, heme

## Abstract

Ferroptosis is a cell death mechanism mediated by iron-dependent lipid peroxidation. Although ferroptosis has garnered attention as a cancer-suppressing mechanism, there are still limited markers available for identifying ferroptotic cells or assessing their sensitivity to ferroptosis. The study focused on biliverdin, an endogenous reducing substance in cells, and examined the dynamics of intracellular biliverdin during ferroptosis using a biliverdin-binding cyanobacteriochrome. It was found that intracellular biliverdin decreases during ferroptosis and that this decrease is specific to ferroptosis amongst different forms of cell death. Furthermore, the feasibility of predicting sensitivity to ferroptosis by measuring intracellular biliverdin was demonstrated using a ferroptosis model induced by the re-expression of the transcription factor BACH1. These findings provide further insight into ferroptosis research and are expected to contribute to the development of cancer therapies that exploit ferroptosis.

## Graphical Abstract

**Graphical Abstract:**
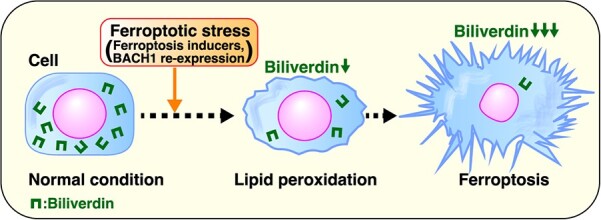

## Abbreviations

ACSL4: acyl-CoA synthetase long-chain family member 4
ANOVA: analysis of variance
BACH1: BTB and CNC homology 1
*Bach1* ^−/−^ mice: *Bach1* knockout mice
BTB: Broad complex, Tramtrack, Bric-a-brac domain
bZIP: basic leucine zipper
CNC: Cap’n’Collar region
DAPI: 4’,6-diamidino-2-phenylindole
DMSO: Dimethyl sulfoxide
Ferr-1: ferrostatin-1
FINs: ferroptosis inducers
GPX4: glutathione peroxidase 4
GSH: glutathione
HO-1 (*Hmox1*): heme oxygenase 1
iMEFs: immortalized MEFs
MDA: malondialdehyde
2-ME: 2-mercaptoethanol
MEFs: mouse embryonic fibroblasts
NAC: N-Acetyl-L-cysteine
PD-L1: programmed cell death ligand-1
PI: Propidium iodide
*Ptgs2*
: prostaglandin-endoperoxide synthase 2
PRDX3: peroxiredoxin 3
RSL3: (1S,3R)-RSL3
SD: standard deviation
SLC7A11: solute carrier family 7 member 11
TNF-α: tumour necrosis factor-α
Z-VAD.FMK: Benzyloxycarbonyl-Val-Ala-Asp (OMe) fluoromethylketone

##  

Ferroptosis is a regulated form of iron-dependent non-apoptotic cell death *(**1**)*. During ferroptosis, lipid hydroperoxides accumulate as a result of the Fenton reaction catalysed by intracellular labile iron, which ultimately leads to membrane rupture and cell death *(**1*–*3**)*. Although ferroptosis has been implicated in the pathogenesis of various diseases including ischemic and neurodegenerative disorders *(**4**,**5**)*, it is also recognized as a cancer suppression mechanism *(**6*–*8**)*, rendering it an intriguing target from these disease perspectives.

The absence of a specific marker for ferroptosis is one of the unresolved issues in its research field. Currently, consensus-based markers for ferroptosis include the expression of the transferrin receptor *(**9**)* and prostaglandin-endoperoxide synthase 2 (*Ptgs2*) *(**10**,**11**)*; however, these markers are not specifically increased only during ferroptosis. Recently, hyperoxidized peroxiredoxin 3 (PRDX3) was reported as a ferroptosis marker *(**12**)*, and its usefulness is anticipated, but at this point, its validation has not been accomplished. In addition, altered expression of key regulatory genes, such as glutathione peroxidase 4 (*GPX4*) *(**10**)*, solute carrier family 7 member 11 (*SLC7A11*) *(**1**)* and acyl-CoA synthetase long-chain family member 4 (*ACSL4*) *(**13*–*15**)*, as well as the detection of intracellular labile iron and glutathione (GSH), which are key substances in ferroptosis regulation *(**16**)*, and lipid peroxidation indicators, such as Liperfluo *(**17**)*, C11-BODYPI (581/591) *(**18**)* and malondialdehyde (MDA) *(**19**)*, has been used as ferroptosis markers. Although each of these markers is effective and sensitive, none are universal, and currently, it is necessary to combine multiple experimental results to evaluate ferroptosis. This current situation may be explained by the fact that ferroptosis is an intricate cell death mechanism with myriad regulatory systems, the full extent of which has yet to be fully elucidated *(**2**,**5**)*. Therefore, further elucidation of the dynamics and effects of intracellular substances in ferroptosis is necessary.

Biliverdin is generated concurrently with labile iron upon heme degradation catalysed by heme oxygenase within cells. It not only reflects the status of intracellular heme and iron metabolism, but also functions as an endogenous reducing agent *(**20**)*. Expression of *Hmox1*, which encodes for heme oxygenase-1 (HO-1), is induced upon ferroptosis *(**21**)*. Therefore, during ferroptosis, it is expected that the dynamics of intracellular biliverdin changes *(**22**)*. Furthermore, intracellular biliverdin may serve as a marker for determining the sensitivity of cells to ferroptosis, assuming that it potentially acts as an inhibitory substance for ferroptosis as a reducing agent *(**20**)*. However, the relationship between ferroptosis and biliverdin, including the dynamics of intracellular biliverdin during ferroptosis, remains unclear.

In this study, the dynamics of intracellular biliverdin during ferroptosis were examined using the cyanobacteriochrome NpF2164g5_BV4, which binds to biliverdin and emits near-infrared fluorescence *(**23**)*. In addition, the potential role of biliverdin as a regulator of ferroptosis or as a sensitivity marker in ferroptosis model cells was also investigated. The results will contribute to an increased understanding of ferroptosis and aid future research and applications associated with this specialized type of cell death.

## Material and Methods

### Mice

The generation of *Bach1*^−/−^ mice on a C57BL/6 J background was described previously *(**24**)*. The mice were bred at the animal facility at Tohoku University and housed under specific pathogen-free conditions. The mice were euthanized by cervical dislocation under anaesthetic inhalation overdose with isoflurane (Cat# 095–06573, Fujifilm-Wako, Osaka, Japan) prior to anatomy. All experiments performed in this study were approved by the Institutional Animal Care and Use Committee of the Tohoku University Environmental & Safety Committee.

### MEFs and cell lines

Mouse embryonic fibroblasts (MEFs) were derived from 13.5-day-old embryos of *Bach1*^−/−^ mice. The method for isolating MEFs was described previously *(**21**)*. MEFs were maintained at 37°C in DMEM (Dulbecco’s Modified Eagle Medium) containing high glucose (Cat#: 11995, Gibco, Carlsbad, CA, USA), 10% FBS (Cat#: 172012, Sigma–Aldrich, St. Louis, MO, USA; or Cat#: 174012, Nichirei, Tokyo, Japan), 1× MEM non-essential amino acids (Cat#: 11140, Gibco), penicillin/streptomycin (100 U/ml and 100 μg/ml each) (Cat#: 15140, Gibco) and 0.1 mM 2-mercaptoethanol (2-ME) (Cat#: M3148, Sigma–Aldrich). For immortalization, MEFs were cultured under 3% oxygen. Once proliferation ceased, the MEFs were maintained without passage until signs of regrowth became apparent. After regrowth, the cultures were replated at a density of 3 × 10^5^ cells per 60-mm culture dish every 3 days under 20% oxygen *(**25**,**26**)*. The generation of control and mouse BACH1–re-expressing immortalized MEFs (iMEFs) was described previously *(**27**,**28**)*. If the medium is changed to the experimental medium (culture medium without 2-ME and penicillin/streptomycin) after washing once with PBS, BACH1–re-expressing iMEFs undergo ferroptosis *(**27**,**28**)*.

HeLa, NIH3T3 and HEK293T cells were purchased from the ATCC (Manassas, VA, USA). Cells were expanded within three passages after purchase and multiple lots were stocked at −80 °C. Mycoplasma contamination check tests were performed using the e-Myco plus Mycoplasma PCR Detection Kit (Cat#: 25237, iNtRON, Seongnam-Si, South Korea). Cells were used at 20 passages or less, but were not independently authenticated. HeLa (Cat#: CCL-2, RRID: CVCL_0030, ATCC) and NIH3T3 (Cat#: CRL-1658, RRID: CVCL_0594, ATCC) cells were maintained at 37°C in culture medium (DMEM with high glucose (Cat#: D5796, Sigma–Aldrich; or Cat#: 044–29765, Fujifilm–Wako) supplemented with 10% FBS (Sigma–Aldrich or Nichirei) and 0.1 mg/ml penicillin/streptomycin (Gibco)) under 20% oxygen for experiments. HEK293T (Cat#: CRL-3216, RRID: CVCL_0063, ATCC) cells were maintained at 37°C in culture medium (DMEM with low glucose (Cat#: D6046, Sigma–Aldrich; or Cat#: 041–29775, Fujifilm–Wako) containing 10% FBS (Sigma–Aldrich or Nichirei) and 0.1 mg/ml penicillin/streptomycin (Gibco)) under 20% oxygen for experiments.

### Reagents

Dimethyl sulfoxide (DMSO), erastin, (1S,3R)-RSL3 (RSL3), Ferrostatin-1 (Ferr-1), N-Acetyl-L-cysteine (NAC), staurosporine, biliverdin and hemin were purchased from Sigma–Aldrich (DMSO, Cat#: D2650; erastin, Cat#: E7781; RSL3, Cat#: SML2234; Ferr-1, Cat#: SML0583; NAC, Cat#: A7250; staurosporine, Cat#: S4400; biliverdin, Cat#: 30891; hemin, Cat#: H9039). SM-164 and benzyloxycarbonyl-Val-Ala-Asp (OMe) fluoromethylketone (Z-VAD.FMK) were purchased from Selleck (SM-164, Cat#: S7089; Z-VAD.FMK, Cat#: S8102; Houston, TX, USA). Tumour necrosis factor-α (TNF-α) was purchased from Merck (Cat#: 654205, Darmstadt, Germany). Erastin, RSL3, Ferr-1, Z-VAD.FMK, staurosporine and SM-164 were dissolved in DMSO and administered to experimental medium. The concentration of DMSO was adjusted amongst each sample. Biliverdin, NAC and TNF-α were dissolved in water and administered to the experimental medium.

### Intracellular biliverdin assessment by flow cytometry

Information regarding the cyanobacteriochrome (Name: NpF2164g5_BV4) that binds to biliverdin was described previously *(**23**)*. To express the cyanobacteriochrome, the plasmid vector pCAG–EGFP–NpF2164g5_BV4, which expresses EGFP, and the cyanobacteriochrome (NpF2164g5_BV4), which binds to biliverdin and emits near-infrared fluorescence, was introduced into cells (*e.g.* HeLa, HEK293T and NIH3T3) with Genejuice (Cat#: 70967, Merck). After 24 h of transfection, erastin, RSL3, biliverdin, Ferr-1, NAC and hemin were administered, followed by analysis using flow cytometry at the indicated time points. Propidium iodide (PI) or 4′,6-diamidino-2-phenylindole (DAPI) staining was used to assess cell death. PI or DAPI was added (1 μg/ml) before flow cytometry. The cells were sorted with FACS Aria II (Becton–Dickinson (BD), Franklin Lakes, NJ, USA), FACS LSRFortessa (BD) or FACS Verse (BD) and analysed using FlowJo software (RRID: SCR_008520, Tree Star, Ashland, OR, USA). Cells that were positive for EGFP were considered cyanobacteriochrome (NpF2164g5_BV4)-expressing cells. Cells that were positive for near-infrared fluorescence in EGFP-positive cells were considered intracellular biliverdin-positive cells. The gating strategy for assessing intracellular biliverdin-positive cells is shown in Supplementary Fig. 1.

### Cell death assessment by flow cytometry

PI, or DAPI, and annexin V staining were used to assess cell death. APC-Annexin V was purchased from BD (Cat#: 550475). The cells were stained with APC-Annexin V according to the manufacturer’s protocols. PI or DAPI was added (1 μg/ml) prior to flow cytometry. The cells were sorted with FACS Aria II (BD), FACS LSRFortessa (BD) or FACS Verse (BD) and analysed with FlowJo software (Tree Star). Cells that were positive for either or both annexin V and PI (or DAPI) were considered dead cells. Conversely, cells that were negative for both annexin V and PI (or DAPI) were considered alive. The gating strategy for assessing alive or dead cells is shown in Supplementary Fig. 2.

### Live cell imaging of decreased intracellular biliverdin

HEK293T cells were plated onto 12-well cell culture plates (CORNING, Corning, NY, USA). DMEM/F12 (no phenol red) (Cat#: 21041, Gibco) including 10% (v/v) FBS (Nichirei) was used as the experimental medium. After transfection with the plasmid vector pCAG-EGFP-NpF2164g5_BV4, erastin (Sigma–Aldrich) was administered 24 h later. Subsequently, cells were incubated at 37°C for 36 h. Serial photography was performed every 10 min for 36 h using a fluorescence microscope (Celldiscover7, Carl ZEISS, Oberkochen, Germany) using a ×20 0.70 NA Plan Apochromat lens. One optical channel and two fluorescent (Near-infrared: NpF2164g5_BV4_chromophore, Green: EGFP) channels were used. The brightness and contrast of optical and fluorescent channels (near-infrared and green) were not modified during the preparation of the figures.

### Statistics

For all experiments, differences in the datasets were considered statistically significant at *P*-values <0.05. Statistical comparisons were performed using one-way or two-way ANOVA, followed by Tukey’s test for multiple group comparisons.

## Results

### Cyanobacteriochrome system detected intracellular biliverdin

**Fig. 1:**
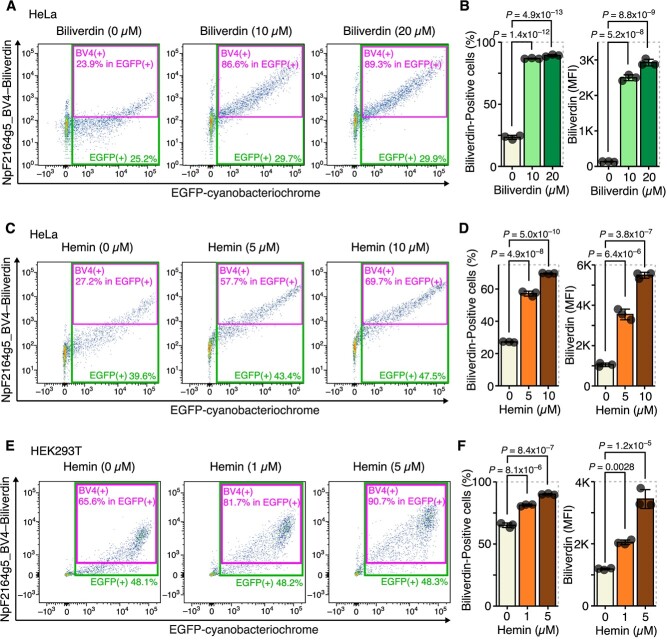
Administration of biliverdin and heme increased intracellular biliverdin. (**A, B**) HeLa cells were transfected with pCAG-EGFP-NpF2164g5_BV4 (a cianobacteriochrome vector) and treated with biliverdin for 24 h. (**A**) Representative flow cytometry images. (**B**) Quantitation of biliverdin-positive cells and mean fluorescence intensity (MFI) in EGFP-positive cells (NpF2164g5_BV4 expressing cells). (**C, D**) HeLa cells were transfected with pCAG-EGFP-NpF2164g5_BV4 and treated with hemin for 24 h. (**C**) Representative flow cytometry images. (**D**) Quantitation of biliverdin-positive cells and MFI in EGFP-positive cells. (**E, F**) HEK293T cells were transfected with pCAG-EGFP-NpF2164g5_BV4 and treated with hemin for 24 h. (**E**) Representative flow cytometry images. (**F**) Quantitation of biliverdin-positive cells and MFI in EGFP-positive cells. Error bars in B, D and F represent the standard deviation (SD). *P*-values in B, D and F were determined by Tukey’s test after a one-way ANOVA. A–F are representative of two independent experiments.

First, we administered biliverdin to cells expressing the cyanobacteriochrome, NpF2164g5_BV4, and determined whether the near-infrared fluorescence emitted by NpF2164g5_BV4 bound to intracellular biliverdin could be detected. The administration of biliverdin resulted in a significant increase in the proportion of cells emitting near-infrared fluorescence (NpF2164g5_BV4-positive cells) amongst those transduced with NpF2164g5_BV4 expression vector (EGFP-positive cells) (Fig. 1A and B, Supplementary Fig. 1). This indicates that administered biliverdin enters the cells and binds to NpF2164g5_BV4, revealing the ability of this system to detect changes in intracellular biliverdin levels. Furthermore, we found that the administration of heme, the source of intracellular biliverdin, also increased intracellular biliverdin (Fig. 1C–F). This suggests that much of the externally administered heme is rapidly broken down by heme oxygenase to supply intracellular biliverdin and iron.

### Intracellular biliverdin is decreased during ferroptosis

**Fig. 2:**
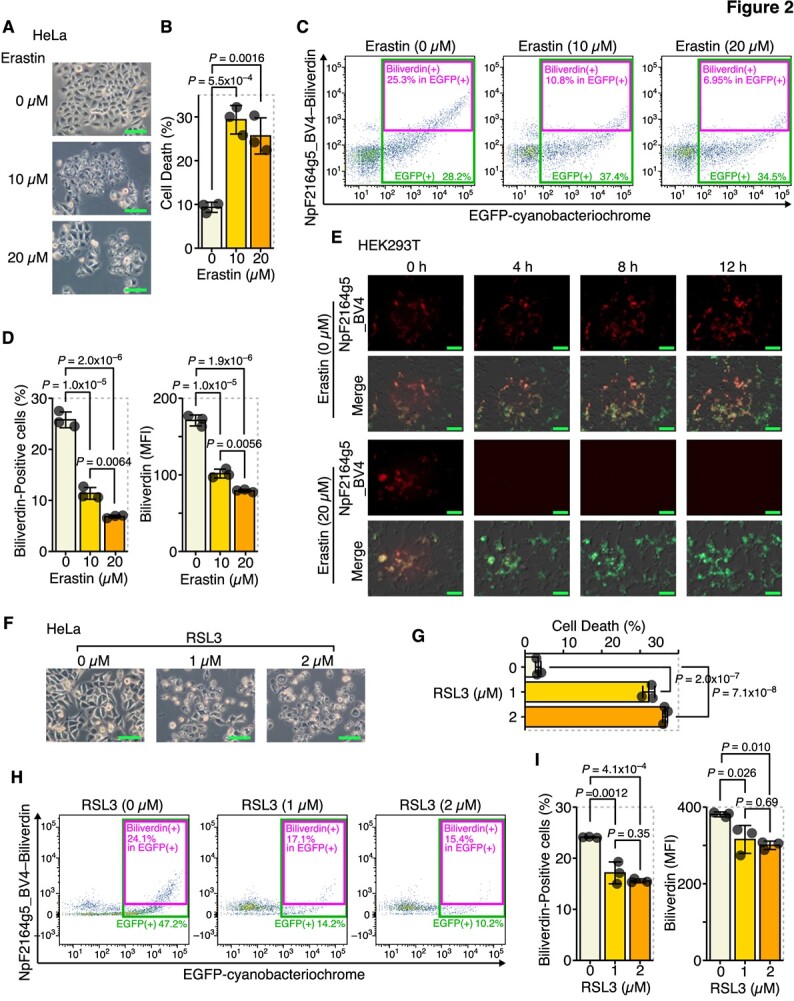
Intracellular biliverdin was decreased during ferroptosis. (**A–D**) HeLa cells were transfected with pCAG-EGFP-NpF2164g5_BV4 and treated with erastin for 24 h. (**A**) Optical microscope images. (**B**) Quantitation of dead cells by flow cytometry. (**C**) Representative flow cytometry images. (**D**) Quantitation of biliverdin-positive cells and MFI in EGFP-positive cells (NpF2164g5_BV4 expressing cells). (**E**) HEK293T cells were transfected with pCAG-EGFP-NpF2164g5_BV4 and treated with erastin for 36 h, with images captured every 10 min. Images of the near-infrared fluorescent channel. Merged images of the optical and fluorescence (green, EGFP; near-infrared, NpF2164g5_BV4) channels. (**F–I**) HeLa cells were transfected with pCAG-EGFP-NpF2164g5_BV4 and treated with RSL3 for 12 h. (**F**) Optical microscope images. (**G**) Quantitation of dead cells by flow cytometry. (**H**) Representative flow cytometry images. (**I**) Quantitation of biliverdin-positive cells and MFI in EGFP-positive cells. Error bars in B, D, G and I represent the SD. *P*-values in B, D, G and I were determined by Tukey’s test after a one-way ANOVA. Scale bars in A, E and F represent 100 μm. A–D and F–I are representative of two independent experiments.

Next, we examined the change in intracellular biliverdin levels upon ferroptosis induction in cells treated with ferroptosis inducers (FINs) using NpF2164g5_BV4. When HeLa cells were treated with erastin, a type 1 FIN (an inhibitor of the cystine/glutamate antiporter System Xc^−^), cell deaths were observed (Fig. 2A and B, Supplementary Fig. 2). When expressing NpF2164g5_BV4 in HeLa cells and examining the change in intracellular endogenous biliverdin levels during ferroptosis, a decrease in intracellular biliverdin was observed upon ferroptosis induction (Fig. 2C and D). Using HEK293T instead of HeLa cells, we confirmed that intracellular biliverdin decreased following erastin treatment (Supplementary Fig. 3A–D). Furthermore, we observed time lapse near-infrared fluorescence derived from NpF2164g5_BV4 bound to intracellular biliverdin. Although cells not treated with erastin did not exhibit a decrease in intracellular biliverdin (Fig. 2E, Supplementary Movies 1A–D), cells treated with erastin exhibited a disappearance of near-infrared fluorescence within a few hours, indicating the rapid consumption of intracellular biliverdin following erastin treatment (Fig. 2E, Supplementary Movies 2A–D). This observation suggests that intracellular biliverdin is consumed before ferroptosis occurs, presumably as a compensatory process for cells to resist undergoing ferroptosis.

Similar experiments were performed using not only erastin but also RSL3, a type 2 FIN (a direct inhibitor of GPX4). This confirmed that cell deaths and a decrease in intracellular biliverdin also occurred upon ferroptosis induction (Fig. 2F–I). A decrease in intracellular biliverdin is considered a general phenomenon of ferroptosis, regardless of the type of FINs.

### Ferroptosis inhibition abolishes a decrease in intracellular biliverdin

**Fig. 3:**
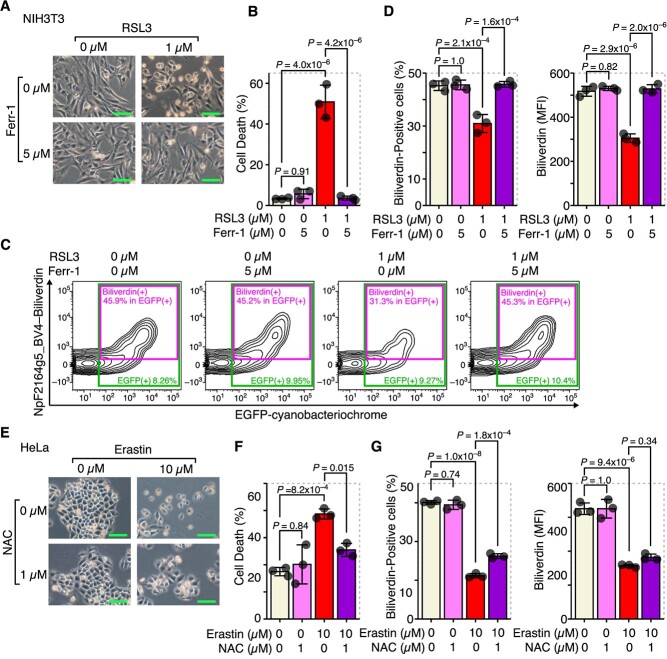
Ferroptosis inhibition abolished the decrease of intracellular biliverdin. (**A–D**) NIH3T3 cells were transfected with pCAG-EGFP-NpF2164g5_BV4 and treated with RSL3 and Ferr-1 for 4 h. (**A**) Optical microscope images. (**B**) Quantitation of dead cells by flow cytometry. (**C**) Representative flow cytometry images. (**D**) Quantitation of biliverdin-positive cells and MFI in EGFP-positive cells (NpF2164g5_BV4-expressing cells). (**E–G**) HeLa cells were transfected with pCAG-EGFP-NpF2164g5_BV4 and exposed to erastin and NAC for 24 h. (**E**) Optical microscope images. (**F**) Quantitation of dead cells by flow cytometry. (**G**) Quantitation of biliverdin-positive cells and MFI in EGFP-positive cells. Error bars in B, D, F and G represent the SD. *P*-values in B, D, F and G were determined by Tukey’s test after a two-way ANOVA. Scale bars in A and E represent 100 μm. A–G are representative of two independent experiments.

Next, we determined whether the decrease in intracellular biliverdin upon ferroptosis is affected when ferroptosis is inhibited by ferroptosis inhibitors. When RSL3-induced ferroptosis was inhibited by ferrostatin-1 (Ferr-1), a specific inhibitor of ferroptosis *(**1**)* (Fig. 3A and B), a decrease in intracellular biliverdin associated with ferroptosis was also reversed (Fig. 3C and D). Furthermore, the reducing agent NAC partially inhibited ferroptosis induced by erastin (Fig. 3E and F), and a decrease in intracellular biliverdin associated with ferroptosis was also partially restored by NAC (Fig. 3G). These results further suggest that a decrease in intracellular biliverdin serves as a marker to reflect the execution of ferroptosis.

### A decrease in intracellular biliverdin is not observed in apoptosis or necroptosis

**Fig. 4:**
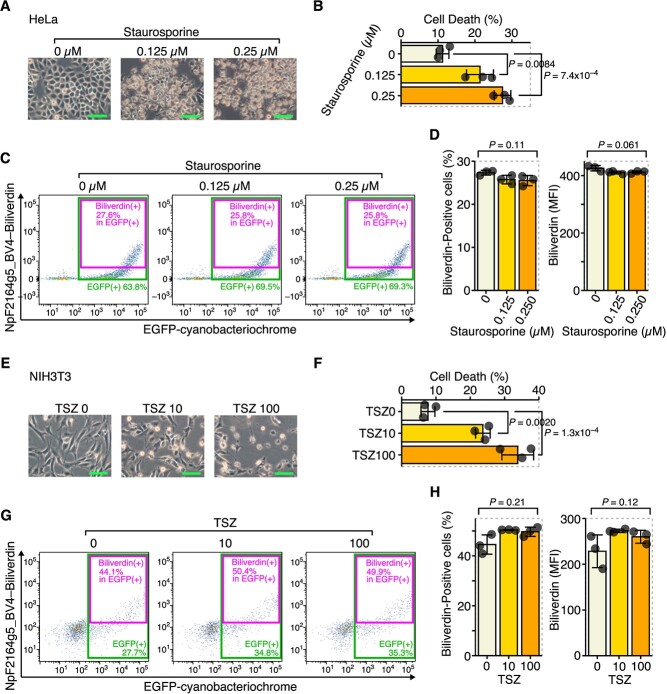
A decrease of intracellular biliverdin was not observed in apoptosis and necroptosis. (**A–D**) HeLa cells were transfected with pCAG-EGFP-NpF2164g5_BV4 and treated with staurosporine for 1 h. (**A**) Optical microscope images. (**B**) Quantitation of dead cells by flow cytometry. (**C**) Representative flow cytometry images. (**D**) Quantitation of biliverdin-positive cells and MFI in EGFP-positive cells (NpF2164g5_BV4-expressing cells). (**E–H**) NIH3T3 cells were transfected with pCAG-EGFP-NpF2164g5_BV4 and treated with TSZ (TNF-α, SM-164 and Z-VAD.FMK) for 24 h. TSZ 0: (TNF-α: 0 ng/ml, SM-164: 0 nM and Z-VAD.FMK: 0 μM), TSZ 10: (TNF-α: 10 ng/ml, SM-164: 100 nM and Z-VAD.FMK: 20 μM), TSZ 100: (TNF-α: 100 ng/ml, SM-164: 100 nM and Z-VAD.FMK: 20 μM). (**E**) Optical microscope images. (**F**) Quantitation of dead cells by flow cytometry. (**G**) Representative flow cytometry images. (**H**) Quantitation of biliverdin-positive cells and MFI in EGFP-positive cells. Error bars in B, D, F and H represent the SD. *P*-values in B and F were determined by Tukey’s test after a one-way ANOVA. *P*-values in D and H were determined by a one-way ANOVA. Scale bars in A and E represent 100 μm. A–H are representative of two independent experiments.

Next, we examined whether a decrease in intracellular biliverdin could also be observed in various forms of cell death other than ferroptosis. When apoptosis was induced by staurosporine or necroptosis was induced by the combination of TNF-α, SM-164 and Z-VAD.FMK (TSZ), cell death was confirmed (Fig. 4A, B, E, F); however, unlike ferroptosis, there was no clear decrease in intracellular biliverdin (Fig. 4C, D, G, H). Based on these results, a decrease in intracellular biliverdin is specific to ferroptosis compared with other forms of cell death.

### Biliverdin acts as a weak ferroptosis inhibitor

**Fig. 5:**
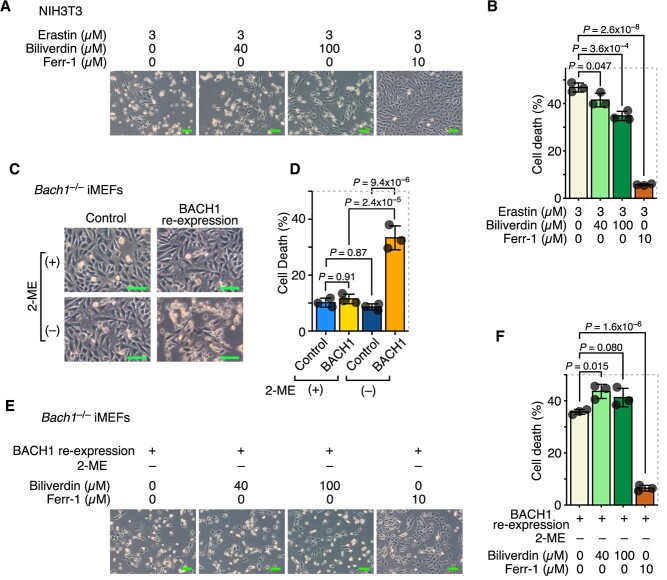
Biliverdin weakly inhibited ferroptosis. (**A, B**) NIH3T3 cells were exposed to erastin, biliverdin and Ferr-1 for 24 h. (**A**) Optical microscope images. (**B**) Quantitation of dead cells by flow cytometry. (**C, D**) The culture medium of BACH1–re-expressing iMEFs was changed and 2-ME was removed. Subsequently, cells were incubated for 24 h. (**C**) Optical microscope images. (**D**) Quantitation of dead cells by flow cytometry. (**E, F**) The culture medium of BACH1–re-expressing iMEFs was changed and 2-ME was removed. Subsequently, cells were treated with biliverdin or Ferr-1 for 24 h. (**E**) Optical microscope images. (**F**) Quantitation of dead cells by flow cytometry. Error bars in B, D and F represent the SD. *P*-values in B and F were determined by Tukey’s test after a one-way ANOVA. *P*-values in D were determined by Tukey’s test after a two-way ANOVA. Scale bars in A, C and E represent 100 μm. A–F are representative of two independent experiments.

Biliverdin may be decreased because it is consumed as an endogenous reducing agent when cells are exposed to ferroptotic stress. Therefore, we examined whether biliverdin suppresses ferroptosis. When biliverdin was administered to cells, erastin-induced ferroptosis was somewhat alleviated by the addition of a relatively high concentration of biliverdin (Fig. 5A and B). However, compared with Ferr-1, the inhibitory effect of biliverdin on ferroptosis was smaller (Fig. 5A and B). We tried to confirm this finding by using another ferroptosis model. The transcription factor BTB and CNC homology 1 (BACH1) strongly promotes ferroptosis by transcriptionally suppressing multiple genes involved in both the GSH synthesis pathway and the labile iron metabolism pathway *(**21**,**29**,**30**)*. Previously, we established a model cell system capable of inducing ferroptosis by re-expressing BACH1 in iMEFs without using FINs *(**27**,**28**)*. Using these cells, we examined whether biliverdin suppressed ferroptosis induced by BACH1 re-expression. These BACH1–re-expressing cells undergo ferroptosis spontaneously when 2-ME is removed from the culture medium; however, ferroptosis is not clearly observed in the presence of 2-ME (Fig. 5C and D) *(**27**)*. Unlike ferroptosis induced by erastin, BACH1–re-expression-induced ferroptosis was not inhibited by biliverdin (Fig. 5E and F). The reason for this is unclear, but the exact profile of ferroptosis may be different depending on the inducing method. This detailed classification presents a challenge for future ferroptosis studies.

### Intracellular biliverdin reflects ferroptosis sensitivity

**Fig. 6:**
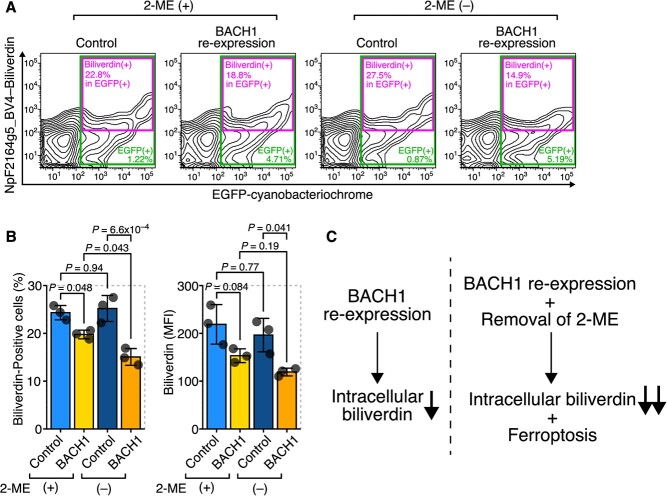
Intracellular biliverdin reflected ferroptosis sensitivity. (**A, B**) BACH1–re-expressing iMEFs were transfected with pCAG-EGFP-NpF2164g5_BV4. 24 h later, the culture medium was changed and 2-ME was removed. Subsequently, cells were incubated for 24 h. (**A**) Representative flow cytometry images. (**B**) Quantitation of biliverdin-positive cells and MFI in EGFP-positive cells (NpF2164g5_BV4-expressing cells). (**C**) Conceptual diagram. The re-expression of BACH1 alone leads to a decrease in intracellular biliverdin levels, suggesting increased sensitivity to ferroptosis. The removal of 2-ME in addition to BACH1 re-expression further decreases intracellular biliverdin levels and induces ferroptosis. Error bars in B represent the SD. *P*-values in B were determined by Tukey’s test after a two-way ANOVA. A and B are representative of two independent experiments.

Finally, we determined whether intracellular biliverdin reflects the potential ferroptosis sensitivity of cells using the ferroptosis model cells with BACH1 re-expression. When measuring intracellular biliverdin in these cells, we found that even before removing 2-ME, the amount of intracellular biliverdin had already been decreased in BACH1–re-expressing cells compared with the control cells, and it was further decreased upon removal of 2-ME (Fig. 6A–C). These results suggest that intracellular biliverdin levels reflect the ferroptosis sensitivity of the cells even before ferroptosis is significantly induced (Fig. 6C). Conversely, measuring intracellular biliverdin levels may enable the determination of ferroptosis sensitivity in cells or tissues and suggests potential future applications in ferroptosis research.

## Discussion

In this study, the intracellular dynamics of biliverdin during ferroptosis were examined using the cyanobacteriochrome NpF2164g5_BV4, which binds to biliverdin and emits near-infrared fluorescence *(**23**)*. During ferroptosis induced by various methods, such as type 1 FINs (*e.g.* erastin), type 2 FINs (*e.g.* RSL3) and BACH1 re-expression (Fig. 2 and 6), a decrease in intracellular biliverdin was observed, which was prevented by ferroptosis inhibitors (Fig. 3). In addition, a decrease in intracellular biliverdin was specific to ferroptosis, as it was not observed in other forms of cell death examined (Fig. 4). These results suggest that intracellular biliverdin levels may serve as a marker for monitoring the execution of ferroptosis. One of the remaining issues concerning this system is that, whilst biliverdin is known to form complexes with iron and other metals within cells *(**31**,**32**)*, it is not yet known whether NpF2164g5_BV4 can detect these biliverdin complexes. In the future, it would be ideal if we could understand the state of biliverdin, including its complexes, through cyanobacteria. Additionally, we used the method of lipofection to overexpress NpF2164g5_BV4 in the present study; however, establishing cell lines that stably express NpF2164g5_BV4 will further contribute to ferroptosis research. Furthermore, if NpF2164g5_BV4 can be readily introduced into tissues of animals, such as mice, it will be possible to examine the relationship between intracellular biliverdin levels and ferroptosis *in vivo*.

Although biliverdin is an endogenous reducing agent, its ferroptosis inhibitory effect was limited in the present study (Fig. 5). In the case of ferroptosis induced by BACH1 re-expression, there was no observed inhibitory effect of biliverdin on ferroptosis at all (Fig. 5F). This could be because, whilst primary mechanism of erastin is considered to be the decrease in glutathione synthesis through the inhibition of System Xc^−^, BACH1 re-expression not only inhibits glutathione synthesis but also causes a more significant increase in intracellular labile iron *(**27**)*. Additionally, recent studies suggest cell sensitivity to ferroptosis can vary widely depending on the profile of lipid peroxides *(**33**,**34**)*, which suggests the existence of complex relationships amongst them. Similarly, the ability of reducing agents to inhibit ferroptosis varies depending on the type of them. Based on the results of the present study, biliverdin was considered a weak reducing agent in terms of its ability to suppress ferroptosis, which is valuable information to consider for future ferroptosis studies. Furthermore, the weak ferroptosis inhibitory effect of biliverdin observed in this study will advance our understanding of the impact of heme and its degrading enzyme, HO-1, on ferroptosis, which has been a long-standing question *(**35**,**36**)*. There are reports suggesting both driver and suppressor roles for HO-1 in ferroptosis *(**37**,**38**)*; however, at present, there are more reports suggesting that HO-1 acts as a driver of ferroptosis (FerrDb database: HGNC 5013 *(**39**)*). Until now, HO-1 is believed to act in both directions, promoting and inhibiting ferroptosis, depending on the balance between the ferroptosis-promoting ability of labile iron and the ferroptosis-inhibiting ability of biliverdin/bilirubin, which are produced by heme degradation *(**35**,**40**)*. However, if the impact of biliverdin on ferroptosis is limited (Fig. 5), it is reasonable to assume HO-1 basically acts as a driver of ferroptosis by degrading heme and supplying labile iron. Further studies are needed to understand the relationship amongst heme, HO-1 and ferroptosis. However, insight gained into the dynamics of intracellular biliverdin during ferroptosis in the present study will help to elucidate the relationship between heme/iron metabolism and ferroptosis.

A remaining issue concerning biliverdin during ferroptosis is related to its localization. Generally, biliverdin is believed to be primarily localized in the mitochondria within the cell, where it is transported to the cytoplasm and reduced to bilirubin by biliverdin reductase *(**41**)*. It is unclear whether biliverdin relocates from the mitochondria to other areas when it decreases during ferroptosis. As shown in Fig. 2E and Supplementary Movies S1 and S2, it is difficult to determine the localization with the overexpressed NpF2164g5_BV4 in this system. The change in biliverdin localization during ferroptosis remains a challenge to be addressed in the future. Furthermore, although information on organelle function during ferroptosis is gradually being accumulated *(**42**)*, the complete picture remains unclear. Understanding the changes in biliverdin localization during ferroptosis could provide valuable insights into the role of organelles during ferroptosis.

Because intracellular biliverdin levels may reflect sensitivity to ferroptosis (Fig. 6), it may have clinical applications such as determining the risk for ferroptosis-related diseases and predicting the efficacy of ferroptosis therapy in malignant tumours. In particular, cancer cells that have developed resistance to molecular-targeted therapy exhibit increased sensitivity to ferroptosis *(**7**,**43**,**44**)*. Therefore, measuring biliverdin levels in tumour tissues during treatment with molecular-targeted therapy may enable the early detection of resistance. Furthermore, as ferroptosis is known to occur in tumour tissues because of immune checkpoint inhibitors *(**45**)*, biliverdin levels in tumours may be able to predict the efficacy of immunotherapy, in addition to assessing programmed cell death ligand-1 (PD-L1) expression *(**46**,**47**)* and DNA mismatch repair status *(**48**)*. Building upon the findings of this study, further advances in the application of ferroptosis to clinical oncology are anticipated.

## Supplementary Material

Web_Material_mvae067
