# Supplementary material for: Intracellular biliverdin dynamics during ferroptosis
Source: J Biochem. 2024 Sep 28;176(6):472–83. doi: 10.1093/jb/mvae067 (PMC11638335; doi:10.1093/jb/mvae067)
Supplement: Web_Material_mvae067 [file web_material_mvae067.zip › Supplementary_Figure_Supplementary_Movie_Legends_mvae067.pdf]

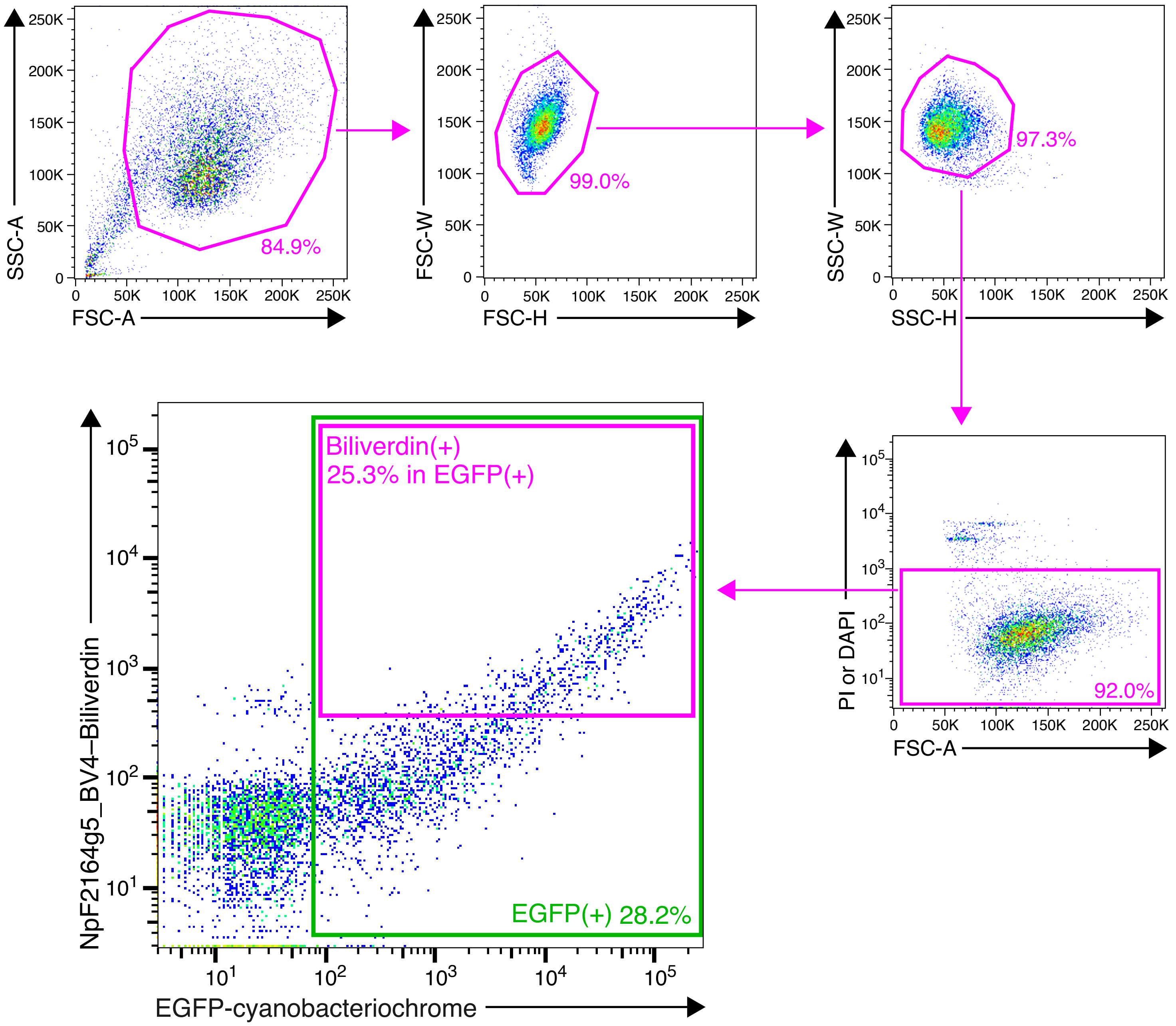

Supplementary Fig. 1: Additional data demonstrating flow cytometry gating (Assessment of intracellular biliverdin). Representative flow cytometry images showing the strategy that was implemented for the sorting of intracellular biliverdin-positive cells. EGFP-positive cells were judged as the cells transfected with a plasmid vector expressing the cyanobacteriochrome (NpF2164g5_BV4) that bind to biliverdin and emit near-infrared fluorescence. NpF2164g5_BV4 (near-infrared fluorescence)–positive cells were judged as the cells abundant in intracellular biliverdin. Representative data was that of HeLa exposed to 0 μM erastin in Fig.2*D*. The similar strategy was implemented in Fig. 1*B*, *D*, and *F*, Fig. 2*D* and *I*, Fig. 3*D* and *G*, Fig. 4*D* and *H*, Fig. 6*B*, and Supplementary Fig. 3*B-D*.

Figure S2

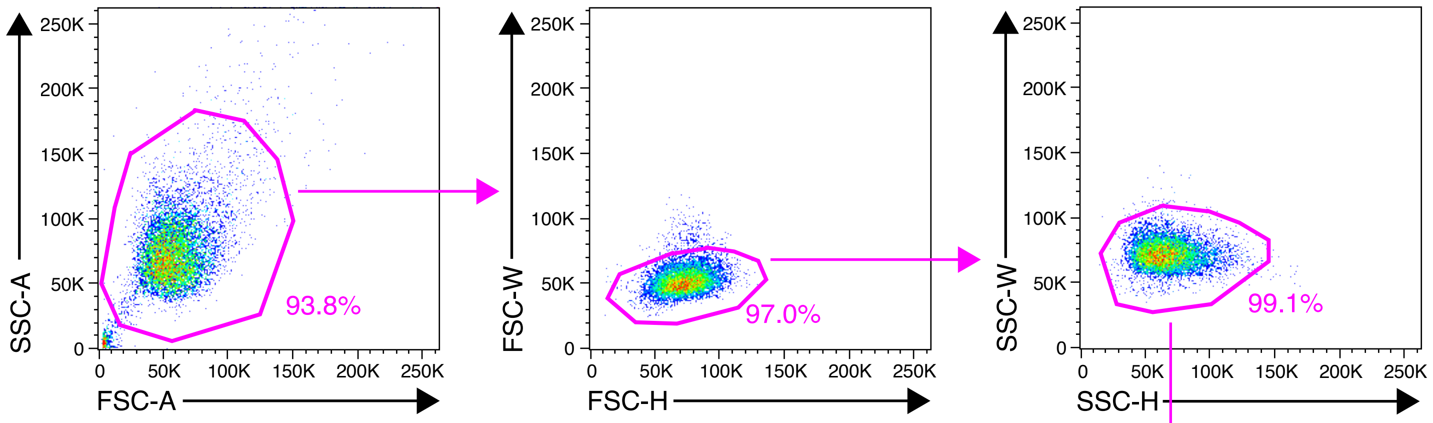

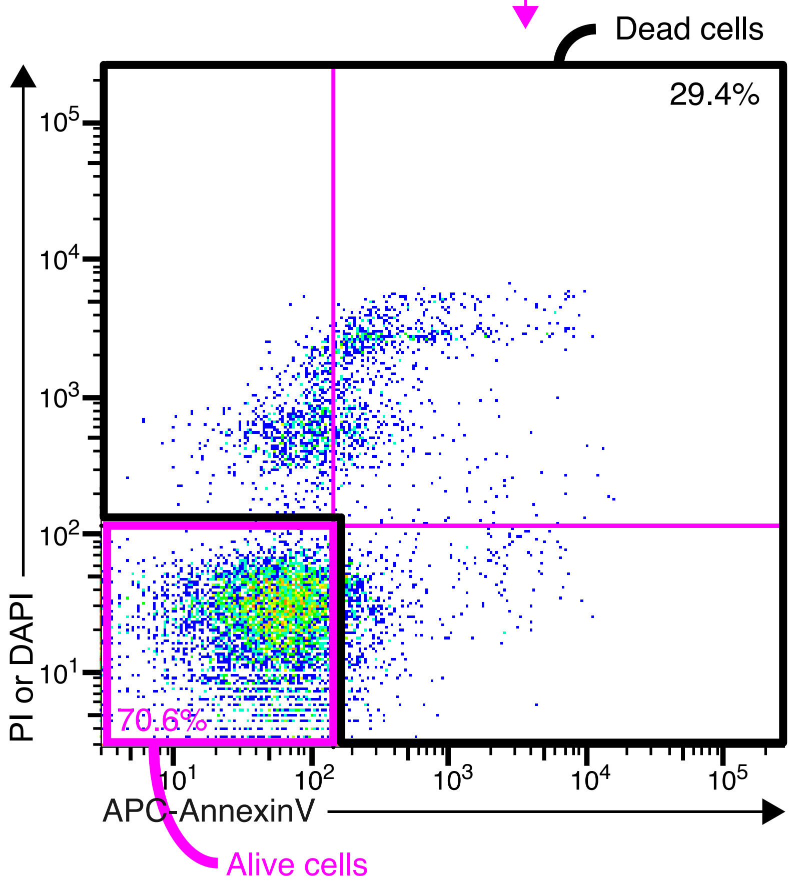

Supplementary Fig. 2: Additional data demonstrating flow cytometry gating (Assessment of cell death). Representative flow cytometry images showing the strategy that was implemented for the sorting of alive cells. PI or DAPI negative, and Annexin V negative cells were judged as alive cells. The other cells were judged as dead cells. Representative data was that of HeLa exposed to 0.25 μM staurosporine in Fig.4*B*. The similar strategy was implemented in Fig. 2*B* and *G*, Fig. 3*B* and *F*, Fig. 4*B* and *F*, and Fig. 5*B*, *D*, and *F*.

Figure S3

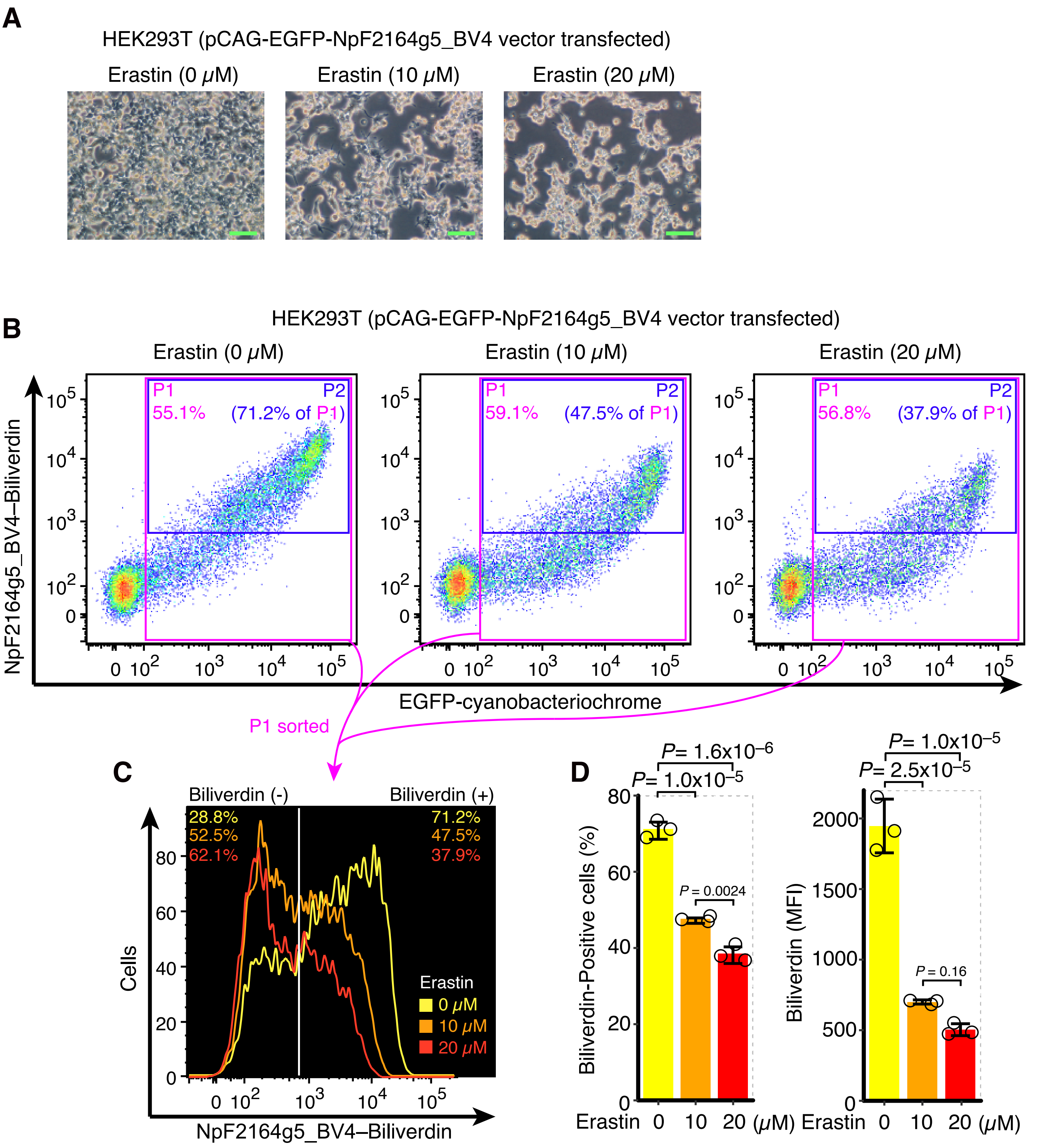

Supplementary Figure 3. Intracellular biliverdin was decreased during ferroptosis. HEK-293T cells were transfected with pCAG-EGFP-NpF2164g5_BV4 (a cianobacteriochrome vector) and treated with erastin for 24 h. (**A**) Optical microscope images. (**B**) Representative flow cytometry images of HEK293T transfected with the vectors, in each density of erastin. (**C**) Histgram of representative samples of transfection-positive cells (P1). (**D**) Quantitation of biliverdin-positive cells and mean fluorescence intensity (MFI). Error bars in *D* represent the standard deviation. *P*-values in *D* were determiened by Tukey's test after a one-way ANOVA. Scale bars in *A* represent 100 μM. (*A-D*) are representative of two independent experiments.

Supplementary movie legends

Supplementary movie 1. Intracellular biliverdin did not decrease under DMSO

(**A-D**) HEK293T cells were transfected with pCAG-EGFP-NpF2164g5_BV4 (a cianobacteriochrome vector) and exposed to DMSO for 36 h, taking pictures every 10 min. (**A**) A movie of near-infrared fluorescent channel. (**B**) A merged movie of optical and fluorescent (green, EGFP; near-infrared, NpF2164g5_BV4) channel. (**C**) Another movie of near-infrared fluorescent channel. (**D**) Another merged movie of optical and fluorescent (green, EGFP; near-infrared, NpF2164g5_BV4) channel. Scale bars in A-D represent 100 μm.

Supplementary movie 2. Intracellular biliverdin decreased under erastin

(**A-D**) HEK293T cells were transfected with pCAG-EGFP-NpF2164g5_BV4 (a cianobacteriochrome vector) and exposed to 20 μM erastin for 36 h, taking pictures every 10 min. (**A**) A movie of near-infrared fluorescent channel. (**B**) A merged movie of optical and fluorescent (green, EGFP; near-infrared, NpF2164g5_BV4) channel. (**C**) Another movie of near-infrared fluorescent channel. (**D**) Another merged movie of optical and fluorescent (green, EGFP; near-infrared, NpF2164g5_BV4) channel. Scale bars in A-D represent 100 μm.
